# DEPRESSION SEVERITY AND OBSTRUCTIVE SLEEP APNEA IN OLDER ADULTS

**DOI:** 10.1093/geroni/igz038.596

**Published:** 2019-11-08

**Authors:** Nicholas C Boston, Ryan Bennett, Nikolas Cirillo, Andrew Solow, Nicole Fornalski, Ashely M Stripling

**Affiliations:** 1 Nova Southeastern University, Davie, Florida, United States; 2 Nova Southeastern University, Fort Lauderdale, Florida, United States; 3 Nova Southeastern University, Fort Lauderdale, United States

## Abstract

Objective: The connection between obstructive sleep apnea and depression in older adults is well documented; however, to date the relationship between severity of these depressive symptoms in this population remains under-explored. As such, the current analysis examined a potential relationship between varying levels of depression severity among older adults with sleep apnea. Participants and Methods: Data was derived from a de-identified database of older adults (age>=65) from the National Alzheimer’s Coordinating Center (NACC). The sample (N=90; 50% female; 97.8% Caucasian; Mage=77 years; SDage=10.4 years) was sorted into three groups using the Neuropsychiatric Inventory Questionnaire (NPI-Q): 1) Mild Depression [n=56], 2) Moderate Depression [n=29], and 3) Severe Depression [n=5]. Results: A univariate analysis revealed an overall significant omnibus effect between sleep apnea and depression severity (F[2,4041])=16.231, p<.001), while controlling for age, race, and sex. Post-hoc comparison found that those with severe depression had significantly higher levels of sleep apnea compared to those with mild (Mdif =-.499, p = .029) and moderate (Mdif =-.597, p = .009). Conclusions: These data support the possible association between depression severity and obstructive sleep apnea. Results may be attributable to two different theories: that low serotonin levels may simultaneously influence depression, respiratory muscle-tone, and sleep disturbance, and that intermittent hypoxia may create a cascade effect of neurovascular pathology resulting in depressive symptoms. Implications of the current findings suggest it may prove beneficial to keep in mind the risks associated with sleep apnea, and more severe depression, should an individual present with either.

